# Immunogenicity Studies of Plant-Produced SARS-CoV-2 Receptor Binding Domain-Based Subunit Vaccine Candidate with Different Adjuvant Formulations

**DOI:** 10.3390/vaccines9070744

**Published:** 2021-07-05

**Authors:** Konlavat Siriwattananon, Suwimon Manopwisedjaroen, Balamurugan Shanmugaraj, Eakachai Prompetchara, Chutitorn Ketloy, Supranee Buranapraditkun, Kittipan Tharakhet, Papatsara Kaewpang, Kiat Ruxrungtham, Arunee Thitithanyanont, Waranyoo Phoolcharoen

**Affiliations:** 1 Research Unit for Plant-Produced Pharmaceuticals, Chulalongkorn University, Bangkok 10330, Thailand; 6176452333@student.chula.ac.th; 2 Department of Pharmacognosy and Pharmaceutical Botany, Faculty of Pharmaceutical Sciences, Chulalongkorn University, Bangkok 10330, Thailand; 3 Department of Microbiology, Faculty of Science, Mahidol University, Bangkok 10400, Thailand; swiboonut@gmail.com (S.M.); arunee.thi@mahidol.edu (A.T.); 4 Baiya Phytopharm Co., Ltd., Bangkok 10330, Thailand; balamurugan.s@baiyaphytopharm.com; 5 Center of Excellence in Vaccine Research and Development (Chula Vaccine Research Center, Chula VRC), Faculty of Medicine, Chulalongkorn University, Bangkok 10330, Thailand; Eakachai.p@chula.ac.th (E.P.); Chutitorn.k@chula.ac.th (C.K.); bsuprane2001@yahoo.com (S.B.); kankittipan12@hotmail.com (K.T.); Kaewpangpapatsara@gmail.com (P.K.); kiat.r@chula.ac.th (K.R.); 6 Department of Laboratory Medicine, Faculty of Medicine, Chulalongkorn University, Bangkok 10330, Thailand; 7 Department of Medicine, Faculty of Medicine, Chulalongkorn University, Bangkok 10330, Thailand

**Keywords:** adjuvant, COVID-19, SARS-CoV-2, receptor-binding domain, plant-based vaccine, subunit vaccine

## Abstract

Due to the rapid transmission of the coronavirus disease 2019 (COVID-19) causing serious public health problems and economic burden, the development of effective vaccines is a high priority for controlling the virus spread. Our group has previously demonstrated that the plant-produced receptor-binding domain (RBD) of SARS-CoV-2 fused with Fc of human IgG was capable of eliciting potent neutralizing antibody and cellular immune responses in animal studies, and the immunogenicity could be improved by the addition of an alum adjuvant. Here, we performed a head-to-head comparison of different commercially available adjuvants, including aluminum hydroxide gel (alum), AddaVax (MF59), monophosphoryl lipid A from *Salmonella minnesota* R595 (mPLA-SM), and polyinosinic-polycytidylic acid (poly(I:C)), in mice by combining them with plant-produced RBD-Fc, and the differences in the immunogenicity of RBD-Fc with different adjuvants were evaluated. The specific antibody responses in terms of total IgG, IgG1, and IgG2a subtypes and neutralizing antibodies, as well as vaccine-specific T-lymphocyte responses, induced by the different tested adjuvants were compared. We observed that all adjuvants tested here induced a high level of total IgG and neutralizing antibodies, but mPLA-SM and poly (I:C) showed the induction of a balanced IgG1 and IgG2a (Th2/Th1) immune response. Further, poly (I:C) significantly increased the frequency of IFN-γ-expressing cells compared with control, whereas no significant difference was observed between the adjuvanted groups. This data revealed the adjuvants’ role in enhancing the immune response of RBD-Fc vaccination and the immune profiles elicited by different adjuvants, which could prove helpful for the rational development of next-generation SARS-CoV-2 RBD-Fc subunit vaccines. However, additional research is essential to further investigate the efficacy and safety of this vaccine formulation before clinical trials.

## 1. Introduction

A newly emerging disease, coronavirus disease 2019 (COVID-19), was first documented from severe pneumonic cases in Wuhan, Hubei province, China by the end of December 2019. A phylogenetic analysis confirmed that severe acute respiratory syndrome virus 2 (SARS-CoV-2) is a new strain of *Betacoronavirus*, which is a causative agent of the COVID-19 epidemic [1,2,3,4]. SARS-CoV-2 has spread to more than 200 countries and territories worldwide with more than 175 million cumulative infected patients and more than 3.7 million deaths that have been reported up to 15 June 2021 [5]. Because of the rapid spread and high mortality rate of COVID-19, effective therapeutic interventions, especially specific anti-viral drugs, neutralizing antibodies, or vaccines are urgently needed for COVID-19 therapy and the prophylaxis of SARS-CoV-2 [6].

Vaccines are biological preparations from disease-associated microorganisms providing active acquired immune responses for preventing particular infectious diseases. Additionally, vaccines allow a more effective strategy that can reduce the severity and mortality from pathogen infection. Thus, the development of affordable SARS-CoV-2-specific vaccines is urgently needed to combat the ongoing pandemic disease [7,8,9,10,11]. Currently, vaccine candidates against SARS-CoV-2 have been developed using various platforms, including inactivated whole virus, plasmid DNA, a viral vector containing the genes for SARS-CoV-2 structural proteins, and recombinant protein-based vaccines [9,12,13,14]. Specifically, the recombinant protein-based subunit vaccines allow for a more secure and well-defined composition of the antigenic agents compared to other types of vaccines and can effectively reduce the high risk of pathogenicity. In addition, the subunit vaccines also provide scalable processes in a cost-effective manner [13,15,16,17,18,19]. Unfortunately, subunit vaccines formulated without an adjuvant still suffer from poor immunogenic profiles in the activation of a heterogeneous population of immune cells and the induction of only partially protective immune responses [18,20,21,22,23].

To date, several substances, including mineral compounds, bacterial products, and oil emulsions, are used as immune enhancers, especially in protein-based subunit vaccines, and demonstrate the ability to induce robust cellular or humoral responses in animal and human experiments. It is important to understand the different adjuvants’ potential to induce a host immune response through different mechanisms of action. For example, mineral salts, particularly aluminum salts (alum) were the first vaccine adjuvants to have been used in human vaccines over the last 70 years. Aluminum salts are economical and safe adjuvants approved by United States Food and Drug Administration (US FDA) and are widely used in several available licensed human vaccines, including hepatitis A and B, diphtheria, tetanus, rabies, and anthrax [24,25]. Alum enhances vaccine efficacy by trapping the soluble antigen, providing a longer duration of immune-cell–antigen interaction and increasing antigen uptake by the antigen-presenting cells (APCs) to the immune cells [24,25,26]. However, aluminum compounds provide inefficient activation of Th1-type immune responses, which are important for viral clearance [20,24,25]. MF59 is an oil-in-water emulsion licensed in Europe for use with pandemic flu vaccines [20,27]. The MF59 adjuvant works through the recruitment and activation of APCs, which can enhance the immune cells at the administration site and lead to the induction of cytokine and chemokine secretions by macrophages and granulocytes. MF59 elicits mixed Th-1 and Th-2 immunities but prefers the induction of Th1-biased immune response [20,24,27,28,29]. Monophosphoryl lipid A (mPLA) is made from a lipopolysaccharide of Gram-negative *Salmonella minnesota* strain R595. mPLA enhances immune responses by activating Toll-like receptor-4 (TLR4), which plays a major role in the induction of innate and adaptive immunities through interactions with B-lymphocyte cells [20,25,30]. The mPLA adjuvant induces strong Th1 cytokines and triggers B cell proliferation in mice, involved in the production of IgG2a isotype antibodies that are a suitable subtype for combating viral pathogens [20,31,32,33,34]. Polyinosinic-polycytidylic acid (poly(I:C)), which is a TLR3 agonist, is currently undergoing various clinical trials against tumors and infectious diseases, especially Human Immunodeficiency Virus (HIV) [20,35,36,37,38]. Poly (I:C) was reported to induce strong Th1 immune responses against several viruses and pathogens [20,36,37,39].

The formulation of a protein-based subunit vaccine incorporating the appropriate immunoadjuvant has become a high-potential strategy to enhance the desired immunogenicity of protein-based SARS-CoV-2 vaccines that could effectively induce robust protective and long-lasting immune responses [20,24,40,41,42]. Hence, the identification of the optimal immunoadjuvants is crucial for the development of a protein-based vaccine against SARS-CoV-2 [12,43]. Several protein-based vaccine candidates for COVID-19 include either potent immunological adjuvants or immunostimulatory molecules to enhance their immunogenicity profiles. The NVX-CoV2373 vaccine from Novavax (USA) carries a SARS-CoV-2 spike glycoprotein adjuvanted with saponin-based Matrix-M [12,44,45]. The squalene-based AS03 adjuvant is used in recombinant protein vaccine candidates developed by Sanofi and Xiamen Innovax Biotech [12,46]. iBio (USA) is using a glucopyranosyl lipid adjuvant and MPL for its SARS-CoV-2 virus-like particles (VLPs) vaccine development [12,47,48]. Soligenix, Inc. (USA) is using CoVaccine HT [12,49], which is an oil-in-water emulsion adjuvant, for its candidate vaccine [50,51]. In addition, our previous study demonstrated that the addition of an alum adjuvant in a plant-produced SARS-CoV-2 RBD-Fc subunit vaccine effectively improves the vaccine efficacy in eliciting immune responses and neutralizing antibodies in both mice and cynomolgus macaques (*Macaca fascicularis*) [11]. Hence, immunoadjuvants are required in vaccine preparations that not only elicit robust immune responses but also possibly reduce the quantity of the antigens and the number of doses required for immune activation.

In this study, we assessed the immunological profile of different adjuvants by utilizing plant-produced SARS-CoV-2 RBD-Fc as a model antigen. Different adjuvants including alum, MF59, mPLA-SM, and poly (I:C) were evaluated for their capacity to enhance the immunogenicity of a plant-produced subunit vaccine. The mice were immunized with different vaccine formulations, including plant-produced SARS-CoV-2 RBD-Fc alone or along with other adjuvants to assess the SARS-CoV-2 RBD-specific antibodies and neutralizing antibodies, as well as cellular immune responses. The mice immunized with PBS were included as negative control. The results suggested that all the tested adjuvants induced both humoral and cellular responses in mice compared to the unadjuvanted control group, which demonstrates the importance of adjuvants in mounting robust immune response for subunit vaccines.

## 2. Materials and Methods

### 2.1. Ethics Statement

Seven-week-old female ICR mice were ordered from Nomura Siam International Co., Ltd. (Bangkok, Thailand) and were maintained in animal facilities with a strictly hygienic conventional system in the Faculty of Medicine, Chulalongkorn University, Thailand. The use of this animal protocol was approved by the Institutional Animal Care and Use Committee, Faculty of Medicine, Chulalongkorn University (permit number: 012/2563).

### 2.2. Protein Expression and Purification

The recombinant plant-produced RBD of SARS-CoV-2 (SARS-CoV-2 RBD-Fc) was selected in order to test the effects of different adjuvants in immunogenic profiles in mice. The expression and purification of SARS-CoV-2 RBD-Fc from *Nicotiana benthamiana* were described in our previous report [11]. Briefly, the codon-optimized synthetic nucleotide sequence of SARS-CoV-2 RBD was engineered by fusing it with the Fc of the human IgG1 at the C-terminus. The recombinant protein was transiently expressed in *N. benthamiana* and purified by using protein A affinity chromatography (Expedeon, UK). The purified plant-produced SARS-CoV-2 RBD-Fc was filtered through 0.22 μm filter (Merck, Boston, MA, USA) prior to animal experiments.

### 2.3. Adjuvants

Alhydrogel 2% (alum), monophosphoryl lipid A from *Salmonella minnesota* R595 (mPLA-SM), AddaVax (MF59), and high-molecular-weight polyinosinic-polycytidylic acid (poly (I:C)) were purchased from InvivoGen (San Diego, CA, USA). All the adjuvants were prepared by following the manufacturer’s protocols in sterile conditions and were formulated with plant-produced SARS-CoV-2 RBD-Fc for mouse immunogenicity studies.

### 2.4. Mice Immunization and Sample Collection

**Figure 1:**
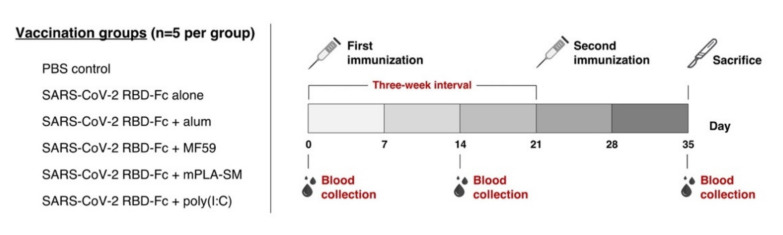
Schematic representation of mouse immunogenicity studies of plant-produced SARS-CoV-2 RBD-Fc and sample collection. Six groups of female ICR mice (*n* = 5 per group) were assigned to receive 2 doses of several vaccine formulations, including 10 μg of plant-produced SARS-CoV-2 RBD-Fc alone or with the aforementioned adjuvants (0.1 mg alum, 10 μg MF59, 10 μg mPLA-SM, and 20 μg poly (I:C)) and PBS for a negative control, on days 0 and 21. Sera were collected on day 0 and 14 days after each vaccination, to investigate vaccine-specific antibody responses and neutralizing activity against SARS-CoV-2. Spleens from the immunized mice were collected on day 35 for evaluating SARS-CoV-2 RBD-specific T cell-mediated response.

An immunogenicity study in mice was performed following our previously optimized protocols [11]. Briefly, female ICR mice were assigned into 6 groups (5 mice per group) for receiving different vaccine formulations via the intramuscular (IM) route with 10 μg of plant-produced SARS-CoV-2 RBD-Fc, either with the presence or absence of immunoadjuvants at the concentration recommended their manufacturers for 2 doses (Table 1) with a 3-week interval, on days 0 and 21. The sera were collected before the first immunization, and 14 days after each vaccination to assess the SARS-CoV-2 RBD-specific total antibody and potent neutralizing antibody responses. Mouse spleens were collected 14 days after the last immunization (day 35) for monitoring the SARS-CoV-2-specific cytokine-producing cells from mouse splenocytes. The schedule of mice immunogenicity is shown in Figure 1.

### 2.5. ELISA

The titers of SARS-CoV-2 RBD-specific antibodies were investigated by ELISA following our previously optimized procedures [11]. Briefly, 96-well plates were coated by Sf9-produced SARS-CoV-2 RBD protein (GenScript, USA) and incubated overnight at 4 °C. The plates were washed by 1xPBST (1xPBS plus 0.05% Tween-20) three times and blocked by 5% skim milk in 1xPBS for 2 h at 37 °C. After three washes with 1xPBST, two-fold serial dilutions of immunized mouse sera, starting at a 1:100 dilution, were added into the wells for 2 h at 37 °C. Subsequently, mouse-specific IgG-detection antibodies, such as goat anti-mouse IgG (HRP) (Jackson ImmunoResearch, USA), goat anti-mouse IgG1 (HRP), and goat anti-mouse IgG2a (HRP) (Abcam, UK) at a dilution of 1:2000 in 1xPBS were incubated at 37 °C for 1 h for the detection of anti-RBD specific total IgG, IgG1, and IgG2a, respectively. The colorimetric reactions were visualized by the addition of a 3,3′,5,5′-tetramethylbenzidine (TMB) solution (Promega, USA) and terminated with 1M H_2_SO_4_.The absorbance at 450 nm (A_450_) was read by an ELISA microplate reader (BMG Labtech, Germany). The titers of mouse antibodies were expressed as the reciprocal of the highest dilution of serum that has A_450_ more than the cut-off [52].

### 2.6. Live SARS-CoV-2 Neutralization Assay

A microneutralization assay was performed to determine the protective efficacy of anti-SARS-CoV-2 neutralizing antibodies. The assay was performed as previously described with some modifications [11]. Briefly, Vero E6 cells (1 × 10^4^ cells/well) were seeded in a 96-well plate and incubated overnight. The serial dilutions of immunized mouse sera were pre-incubated with a 100TCID_50_ of live SARS-CoV-2 for 1 h at 37 °C before transfer to the 96-well tissue culture plates. After 48 h incubation, the SARS-CoV-2 infected cells were detected by using a 1:5000 dilution of anti-SARS-CoV-2 nucleocapsid mAb (SinoBiological, USA) and 1:2000 dilution of goat anti-rabbit IgG antibody (HRP) (Dako, Denmark). The absorbance was read at 450 nm and 620 nm using a Sunrise microplate reader (Tecan, Switzerland). The neutralizing antibody titers were expressed as the reciprocal of the highest dilution of serum that has absorbance differences between 450 and 620 nm (A_450_–A_620_) over the cut-off [53].

### 2.7. Quantitative Measurement of Murine T Lymphocyte Responses

SARS-CoV-2-specific murine T lymphocytes were quantified from mouse splenocytes by following our previous protocols [11] using a mouse IFN-γ ELISpot kit (Mabtech, Sweden). The mouse splenocytes were prepared and pre-activated by SARS-CoV-2 peptides (BioNet-Asia, Thailand, and Mimotopes, Australia) and then incubated with a capture antibody, anti-mouse IFN-γ (AN18) mAb (Mabtech, Sweden), for 3 h at 37 °C. The IFN-γ secreting cells were detected by using a mouse IFN-γ-specific mAb with a biotinylated conjugate (Mabtech, Sweden), followed by incubation with streptavidin-alkaline phosphatase for 1 h at RT. The enzymatic reactions were developed by using a 5-bromo-4-chloro-3-indolyl-phosphate/nitro blue tetrazolium (BCIP/NBT) solution and terminated by rinsing with water. The spots were counted and analyzed by an ELISpot reader (ImmunoSpot Analyzer, USA). The results were expressed in terms of spot-forming cells (SFCs)/10^6^ splenocytes.

### 2.8. Statistical Analysis

The results were analyzed using Prism 8.0 (GraphPad software, USA). Data are expressed as mean ± standard deviation (SD). Dunnett’s tests of multiple comparison were also carried out. Values of *p* < 0.05 were considered statistically significant.

## 3. Results

### 3.1. Adjuvanted SARS-CoV-2 RBD Protein Induced the Highest Titers of IgG Antibody Responses in Immunized Mice

**Figure 2:**
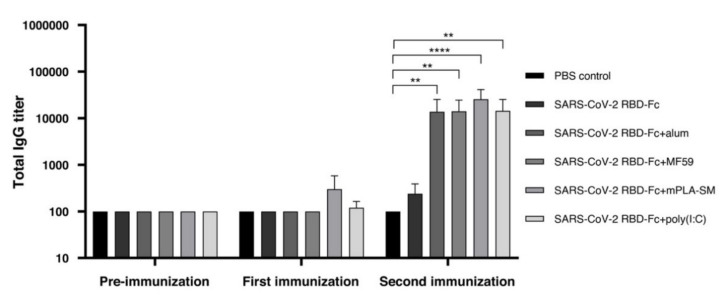
The SARS-CoV-2 RBD-specific total IgG antibody response elicited by mice immunized with different plant-produced SARS-CoV-2 RBD-Fc vaccine formulations. The titers were expressed as endpoint titers, which were analyzed by indirect ELISA using commercial SARS-CoV-2 RBD produced from Sf9 cells as a capture antigen and detected with goat-anti mouse IgG-HRP conjugated antibody. The immunological data were presented as mean ± SD of the endpoint titers from five mice in each vaccination group (*n* = 5). * *p* < 0.05; ** *p* < 0.01; *** *p* < 0.001; ****p < 0.0001.

Our previous study showed that the plant-produced SARS-CoV-2 RBD-Fc protein alone without an adjuvant was immunogenic, inducing SARS-CoV-2 RBD-specific IgG antibody responses in mice, whereas the addition of an adjuvant greatly enhanced its immunogenicity [11]. Subsequently, the ability of different adjuvants, such as alum, mPLA-SM, MF59, and poly (I:C), to induce IgG antibody responses in mice were evaluated. To accomplish this, ICR mice were intramuscularly vaccinated with two doses of plant-produced SARS-CoV-2 RBD-Fc in the presence and absence of the mentioned adjuvants (Table 1), and the mice sera were collected on day 0 and 14 days post each immunization; the mouse antibody titers were evaluated by ELISA (Figure 1). The detection of anti-RBD-specific IgG was slightly increased in mice immunized with plant-produced SARS-CoV-2 RBD-Fc alone, whereas the levels of total IgG responses were significantly increased in mice immunized with plant-produced SARS-CoV-2 RBD-Fc along with the tested adjuvants compared to the control group (Figure 2). Notably, mPLA-SM adjuvanted with plant-produced SARS-CoV-2 RBD-Fc showed a slight increase in SARS-CoV-2 RBD-specific IgG responses after first immunization, whereas no statistical differences in total IgG antibody response were observed between the different adjuvants tested after the second immunization. In addition, mice immunized with PBS failed to induce specific antibody responses (Figure 2).

### 3.2. SARS-CoV-2 RBD-Specific IgG1 and IgG2a Subtype Responses in Mice Immunized with RBD-Fc with Different Adjuvants

**Figure 3:**
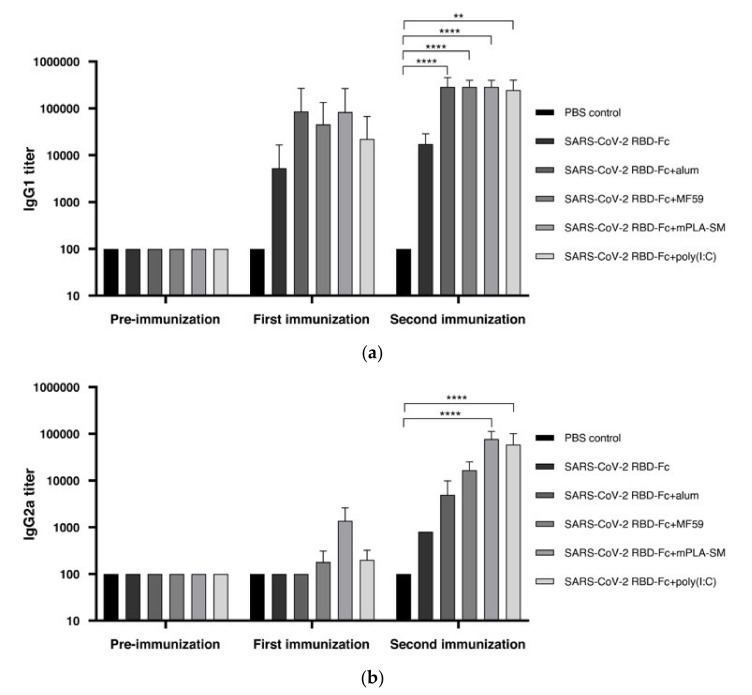
The endpoint titers of SARS-CoV-2 RBD-specific IgG1 (**a**) and IgG2a (**b**) detected in the immunized sera, which were collected on days 0, 14, and 35 and analyzed by indirect ELISA using commercial SARS-CoV-2 RBD produced from Sf9 cells as a capture antigen and the mouse-specific detection antibodies, including goat-anti mouse IgG1-HRP and goat anti-mouse IgG2a-HRP antibody, respectively. The immunological data were presented as mean ± SD of the endpoint titers from five mice in each vaccination group (*n* = 5). * *p* < 0.05; ** *p* < 0.01; *** *p* < 0.001; **** *p* < 0.0001.

The levels of SARS-CoV-2 RBD-specific IgG1 and IgG2a subtypes were also comparatively evaluated, which can refer to Th2 and Th1 cellular responses in mice, respectively. As shown in Figure 3a,b, the plant-produced SARS-CoV-2 RBD-Fc alone could induce detectable IgG1 and IgG2a responses in mice after the second immunization. Specifically, all of the tested adjuvants significantly enhanced the levels of IgG1 responses in mice (Figure 3a), whereas both mPLA-SM and poly (I:C) showed significant increases in IgG2a subtype responses in mice immunized with two doses of plant-produced SARS-CoV-2 RBD-Fc protein, compared to the control (Figure 3b).

### 3.3. Protective Efficacy against Live SARS-CoV-2 In Vitro

**Figure 4:**
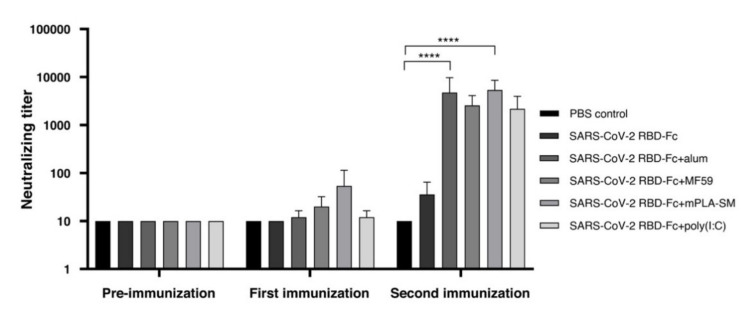
The neutralizing titers detected in mouse sera, which were elicited by mice immunized with several plant-produced SARS-CoV-2 RBD-Fc vaccine formulations against SARS-CoV-2. The in vitro neutralizing responses were assessed by a microneutralization assay using Vero E6 cells. The infected cells were detected by an anti-SARS-CoV-2 nucleocapsid mAb and a goat anti-rabbit IgG-HRP antibody. The immunological data were presented as mean ± SD of the endpoint titers from five mice in each vaccination group (*n* = 5). * *p* < 0.05; ** *p* < 0.01; *** *p* < 0.001; **** *p* < 0.0001.

We further assessed the neutralizing activity of immunized sera using an in vitro microneutralization assay using Vero E6 cells. The results showed that no detectable neutralizing antibody responses were observed in mice immunized with plant-produced SARS-CoV-2 RBD-Fc alone after first immunization, whilst the neutralization titer was slightly increased with a dilution of 1:40 after the second boost. The plant-produced SARS-CoV-2 RBD-Fc alone was capable of eliciting neutralizing antibodies to an extent after two doses of the antigen (Figure 4). In contrast, the addition of all tested adjuvants improves its immunogenicity profiles and elicit significantly higher neutralizing titers, which were observed after the second vaccination, compared to the mice immunized with PBS and plant-produced SARS-CoV-2 RBD-Fc alone at a dilution of approximately 1:5000 (Figure 4). However, there were no significant differences in the neutralizing titer between different tested adjuvants.

### 3.4. IFN-γ-Expressing T Cell Immune Responses Induced by Plant-Produced SARS-CoV-2 RBD-Fc Protein

**Figure 5:**
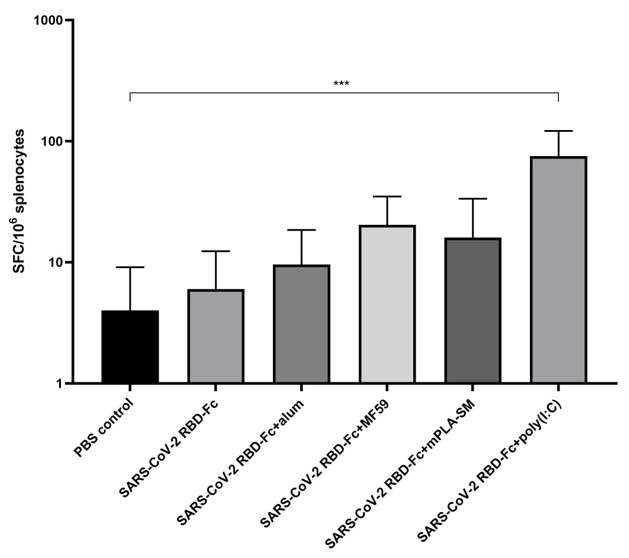
The levels of SARS-CoV-2 RBD-specific IFN-γ-producing T cells expressing from mouse splenocytes immunized with different plant-produced SARS-CoV-2 RBD-Fc vaccine formulations. The IFN-γ expression levels were quantified by mouse ELISpot assay. Data are expressed as mean ± SD of the spot-forming cells (SFCs)/10^6^ splenocytes from five mice in each vaccination group (*n* = 5). * *p* < 0.05; ** *p* < 0.01; *** *p* < 0.001; **** *p* < 0.0001.

The SARS-CoV-2 RBD-specific T cell immunity, which was induced by different formulations of plant-produced SARS-CoV-2 RBD-Fc, was evaluated. Additionally, we appraised a comparison of the ability of immunoadjuvants, including alum, MF59, mPLA-SM, and poly (I:C), to enhance IFN-γ expression. To accomplish the comparison of T cell-mediated immune responses, the mouse spleens were collected on day 35 (14 days after second immunization), and the levels of SARS-CoV-2 RBD-specific IFN-γ expression were assessed from mouse splenocytes by IFN-γ ELISpot assay. Plant-produced SARS-CoV-2 RBD-Fc itself can be capable of inducing cellular immunity, which can be exhibited by the expression level of IFN-γ, compared with the control (Figure 5). However, the formulation of antigens with the tested adjuvants, such as alum, MF59, mPLA-SM, and poly (I:C), increase the frequency of IFN-γ secreting cells when compared with mice immunized with plant-produced SARS-CoV-2 RBD-Fc alone or a control group. The poly (I:C) elicited significantly higher levels of IFN-γ expression in comparison to the PBS control, whereas no significant difference was observed between the adjuvanted groups (Figure 5). Poly (I:C) enhanced vaccine-specific cellular immunity in mice and showed the highest level of specific IFN-γ expression at the level of 75 spots forming cell (SFCs)/10^6^ splenocytes (Figure 5).

## 4. Discussion

The pandemic of COVID-19 caused by SARS-CoV-2 has been posing a severe threat to global health and the economy. Hence, the development of a safe and effective COVID-19 vaccine, which can elicit potent immune responses and protective efficacy against SARS-CoV-2 infection, is required to control the outbreak [7,8,9,10,54]. Comprehensive understanding on SARS-CoV-2 infection confirmed that the S protein containing RBD interacts with ACE2 receptor on the host cell surface, which is an initial step in the viral entry into host cells [2,3,7,54,55,56,57,58]. Particularly, RBD contains the dominant conformational epitopes, which shows great immunogenicity and effectively elicits potent neutralizing antibodies and protective efficacy against SARS-CoV-2 infection in pre-clinical studies, suggesting a vaccine consisting of RBD is a promising target for COVID-19 vaccine development [11,59,60,61,62].

Compared with the other vaccine platforms, recombinant protein-based subunit vaccines provide high safety profiles with no risk of the vaccine triggering disease due to the lack of live virion components. Hence, the protein-based subunit vaccines are considered an attractive vaccine platform and suitable for people, especially immunocompromised patients. Additionally, protein subunit vaccines are easy to produce by utilizing recombinant protein techniques and are relatively stable in comparison to whole virus and viral-vectored vaccines [9,13,19,63,64]. However, the absence of the immunomodulatory components associated with viral particles provides the poor immunogenicity induced by protein subunit immunogens [20,21,22,43,65,66]. Formulation of vaccine antigens with potent immunologic adjuvants or immunostimulatory molecules is an attractive strategy for enhancing the immunogenicity and improving the quality of specific immune responses induced by vaccine antigens [20,24,28,41,65,66]. Additionally, immunologic adjuvants facilitate subunit vaccine development by several beneficial mechanisms of action, such as preventing the rapid degradation of proteins in vivo, enhancing the dose effectiveness of vaccines, and inducing the production of cytokines, which favor the development of T-helper I and II responses to vaccine antigens [22,25,65,66,67,68,69]. The identification and selection of immunologic adjuvants are thus important for successful protein-based subunit vaccine development.

The ability of the plant expression system to produce diagnostic reagents, vaccine antigens, and monoclonal antibodies is well-documented [70,71,72,73,74,75,76,77,78,79,80]. Our group has recently developed a plant-produced subunit vaccine containing SARS-CoV-2 RBD domain fused with the Fc region of human IgG (SARS-CoV-2 RBD-Fc). which showed high affinity binding to commercial ACE2 protein in vitro and elicited high level of immune responses and neutralizing antibodies in mice and cynomolgus macaques (*Macaca fascicularis*). Plant-produced SARS-CoV-2 RBD-Fc also induced cell-mediated immune responses, as judged by its ability to induce the IFN-γ expression observed in mouse splenocytes and monkey peripheral blood mononuclear cells (PBMCs). The results also showed that the addition of an alum adjuvant could enhance the immunogenicity of plant-produced SARS-CoV-2 RBD-Fc antigens [11]. However, the identification and selection of an optimal adjuvant could further improve vaccine efficacy and allow more desirable immunological profiles of plant-produced SARS-CoV-2 RBD-Fc subunit vaccine for combating SARS-CoV-2.

In fact, few adjuvants have been approved for use in licensed human vaccines, including alum, which was used in various human vaccines [20,25], and MF59, which was used in influenza vaccines [20,25,27], and in combination adjuvant systems, such as AS01 and AS04 containing mPLA, used in malaria, and hepatitis B (HBV) and human papilloma virus (HPV) vaccines [20,25,30]. In addition, there are also several experimental adjuvants, which are recruiting in clinical trials, especially poly (I:C), which are used in formulations for H5N1 influenza and cancer vaccines currently in phase III and I/II clinical stages, respectively [20,35,38]. Immunologic adjuvants induce diverse mechanisms of action, which are critical for adjuvant selection and vaccine design to elicit appropriate immune responses against specific viral pathogens. Aluminum salts increase the stability and immunogenicity of vaccine antigens by antigen adsorption onto alum particles and facilitate the gradual release of antigens, resulting in the extension of local pro-inflammatory induction at the administration site [20,25,81]. Alum-based vaccines induce innate immunity by promoting antigen presentation by human macrophage, leading to the upregulation of MHC class II and co-stimulatory molecules, especially CD40, CD 80, and CD86. Additionally, alum salts act on interleukin-4 (IL-4) secretion, involved in the stimulation of Th2 responses and the production of IgG1 and IgE immunoglobulins, and on eosinophils, suggesting that alum is a strong inducer of Th2 responses in mice and humans [20,24,27,81,82,83]. However, alum salts suppress the protective Th1-associated immunity, which is significant for combating intracellular pathogens [20,24,25]. MF59, an oil-in-water emulsion-based adjuvant, also induces local immunostimulatory molecules at the injection site, upregulating several cytokines and chemokines and providing for the recruitment of immune cells in the muscle [20,24,25,27,84]. MF59 preferentially stimulates Th1-based immunity or mixed Th1/Th17 and Th1/Th2 responses, which are required for intracellular pathogen protection [25,28,84]. Interestingly, MF59 showed more effective adjuvanticity in the induction of cell-mediated immunity against influenza virus in human populations, compared to alum salts [24,43]. mPLA allows the preferential recruitment of TIR-domain-containing adapter-inducing interferon-β (TRIF) responding to the activation of TLR4, which leads to decreasing the induction of inflammatory cytokines [31,32,33,85]. MPL promotes monocytes to produce IL-10 and induces IFN-γ expression from NK cells and CD8^+^ T cells in mice [20,43,86]. mPLA is a Th1 inducer, which increases Th1 immune responses in combination with an alum adjuvant in AS04 and results in IgG2a-subtype-eliciting in mice [20,34]. In addition, mPLA also improves cell-mediated immunity against hepatitis B antigens in comparison to vaccine formulated with alum alone [24,67]. Poly (I:C) activates immune responses through endosomal TLR3and cytosolic sensors, including melanocyte differentiation-associated 5 (MDA-5) and retinoic acid-inducible protein I (RIG-I), resulting in MHC class I expression and type I IFN production. Poly (I:C) promotes the cross-presentation of antigens to CD8^+^ T lymphocytes and induces the production of several chemokines and cytokines in murine respiratory tissues, which generates the activation of dendritic cells, macrophages, and neutrophils [20,39,87,88,89]. Poly (I:C) also stimulates a strong type I interferon, type III interferon, and Th1 cytokine responses, which results in IgG2a subtype eliciting in mice, which is required for the clearance of viruses and other pathogens [20,36,37,39,90,91].

In this study, a head-to-head comparison of different adjuvants was performed using the plant-produced SARS-CoV-2 RBD-Fc as the target antigen. Plant-produced SARS-CoV-2 RBD-Fc was formulated with various adjuvants, including alum, MF59, mPLA-SM, and poly (I:C), followed by a comparison of immunological profiles in terms of SARS-CoV-2-specific IgG response, neutralizing antibodies, and IFN-γ-expressing cells from murine splenocytes, in order to assess SARS-CoV-2-specific T-cell-mediated responses [11].

Our results clearly showed that the addition of immunoadjuvants could increase SARS-CoV-2 RBD-specific total antibody responses and neutralizing capacity compared to the mice immunized with the SARS-CoV-2 RBD-Fc antigen alone. All tested adjuvants showed high humoral immune responses, particularly total IgG (Figure 2) and IgG1 subtype (Figure 3a) responses, suggesting that these adjuvants could elicit favorable humoral responses. Interestingly, mPLA-SM and poly (I:C) induce significant levels of IgG2a subtype, which can refer to Th1-biased responses in plant-produced SARS-CoV-2 RBD-Fc vaccination, as confirmed by IgG2a titers with more than 51,200-fold dilution of immunized sera after second immunization, whereas alum and MF59 provided the Th2-biased immune responses (Figure 3b). IgG2a subtype antibody plays a dominant role in viral clearance with high-affinity interaction of activatory Fc receptors providing effective Fc-receptor-mediated immune responses [43,92,93]. The activation of the IgG2a subtype could also increase the survival rate from viral infections and enhance the efficacy of vaccination and viral protection in vivo [92,94,95,96]. Plant-produced SARS-CoV-2 RBD-Fc formulated with tested adjuvants increased their neutralizing ability against SARS-CoV-2 in vitro in comparison to mice immunized with SARS-CoV-2 RBD-Fc alone after the second boost (Figure 4). Upon SARS-CoV-2 RBD-Fc immunization, we observed that alum and mPLA-SM could significantly induce neutralizing antibody responses, which inhibited SARS-CoV-2 infection in vitro in comparison to the PBS control group after the second boost (Figure 4). However, alum-induced Th1 (IgG2a) responds inefficiently, hence the co-administration of alum combining with other Th1 adjuvants is needed [24,25,43]. In ELISA, there was no or less detectable RBD-specific IgG response observed in the mice immunized with SARS-CoV-2 RBD-Fc either with or without adjuvants after the first immunization (Figure 2), whereas titers were observed in IgG1 (Figure 3) after the first immunization. This might be due to the high background values caused by the pre-bleed sera with the different antibodies used for analysis of IgG and IgG subtypes. However, the IgG titer values were consistent with the neutralizing antibody titer analyzed by in vitro neutralization assay. A comparison of vaccine-specific IFN-γ expression induced by plant-produced SARS-CoV-2 with different tested adjuvants was also evaluated using mouse splenocytes. The results showed that poly (I:C) elicits significant level of IFN-γ-expressing T cells over the control group, whereas no statistical difference was observed between the adjuvanted groups (Figure 5). IFN-γ is secreted from T-lymphocytes, especially CD4^+^ T cells, which promotes the activation of macrophage and innate immunity and enhances antigen presentation mediating antiviral activity [97,98,99]. IFN-γ also induces the differentiation of CD4^+^ to Th1 responses, which are required for pathogen clearance, indicating that IFN-γ plays a critical role in vaccine-induced immunity [100,101,102]. Based on these results, all of the adjuvants effectively elicited both humoral and cell-mediated immune responses against the viral infection in mice compared to control. However, further studies are needed to investigate the desired effects and efficacy of the tested adjuvants.

## 5. Conclusions

In summary, our data demonstrated that all tested adjuvants could improve the immunogenicity of plant-produced SARS-CoV-2 RBD-Fc subunit vaccines by eliciting robust humoral and cellular responses against SARS-CoV-2. However, additional research is essential to further investigate the efficacy and safety of all of the tested adjuvants to support this preliminary data. To our knowledge, this is the first report of a non-clinical comparison of different immunoadjuvants to enhance the immunogenicity of plant-produced protein-based subunit vaccine against SARS-CoV-2. The present study provides a foundation to identify the optimal adjuvants for plant-produced subunit vaccines against SARS-CoV-2 in order to develop them as a potential candidate vaccine formulation for clinical evaluation.

## Figures and Tables

**Table 1:** Study groups in mice immunized with a plant-produced SARS-CoV-2 RBD-Fc vaccine formulated with different adjuvants.

Group	Antigen Content	Adjuvant Content	Immunized Volume (μL)
1	PBS control	-	50
2	10 μg SARS-CoV-2 RBD-Fc	-	50
3	10 μg SARS-CoV-2 RBD-Fc	0.1 mg alum	50
4	10 μg SARS-CoV-2 RBD-Fc	10 μg MF59	50
5	10 μg SARS-CoV-2 RBD-Fc	10 μg mPLA-SM	50
6	10 μg SARS-CoV-2 RBD-Fc	20 μg poly (I:C)	50

## Data Availability

The data are available under reasonable request to the corresponding author.
